# An examination of the emerging field of community paramedicine: a national cross-sectional survey of community paramedics

**DOI:** 10.1186/s12913-023-09537-x

**Published:** 2023-05-23

**Authors:** Chinyere Mma Okoh, Leticia R. Moczygemba, Whitney Thurman, Carolyn Brown, Christopher Hanson, James O. Baffoe

**Affiliations:** 1 Health Outcomes Division, The University of Texas at Austin College of Pharmacy, 2409 University Ave, #A1900, Austin, TX 78712 USA; 2 The University of Texas at Austin School of Nursing, 1710 Red River St, Austin, TX 78712 USA; 3 TMF Health Quality Institute, 3107 Oak Creek Dr Ste. 200, Austin, TX 78727 USA

**Keywords:** Community paramedicine, Professional identity, Interprofessional collaboration, Role clarity, Role readiness, COVID-19

## Abstract

**Background:**

Community Paramedicine is an evolving community-based model that expands paramedic roles from emergency and transport care to a focus on non-emergent and preventive health services tailored to local community needs. Though community paramedicine is a growing field and acceptance is gradually increasing, there is limited information on community paramedics (CPs) perceptions of their expanded roles. The study’s aim is to assess CPs’ perceptions about their training, roles, role clarity, role readiness, role satisfaction, professional identity, interprofessional collaboration, and the future of the community paramedicine care model.

**Methods:**

Using the National Association of Emergency Medical Technicians-mobile integrated health (NAEMT-MIH) listserv, a cross-sectional survey was conducted in July/August 2020 using a 43-item web-based questionnaire. Thirty-nine questions evaluated CPs’ training, roles, role clarity, role readiness, role satisfaction, professional identity, interprofessional collaboration, and program/work characteristics. Four open-ended questions examined perceptions of the future of community paramedicine care models and challenges/opportunities encountered during the COVID-19 pandemic. Data was analyzed using Spearman’s correlation, Wilcoxon Mann–Whitney U, and Kruskal–Wallis tests. Open-ended questions were analyzed using qualitative content analyses.

**Results:**

Responses from fifty-seven CPs were analyzed. Most (80%) completed didactic and/or clinical training. Nearly all respondents (96.5%) performed health assessments; only 38.6% administered vaccines. Overall, participants were neutral about their role readiness with a mean score of 3.3/5.0. The mean role clarity was 15.5 (range 4–29; higher scores = higher clarity), professional identity was 46.8 (range 30–55; higher scores = higher identity), role satisfaction was 4.4/5 with 5 = very satisfied, and interprofessional collaboration was 9.5/10 (10 = very important). Role clarity training (rho = 0.4, *p* = 0.0013) and higher interprofessional collaboration (rho = 0.4, *p* = 0.0015) were found to be significantly associated with the enhancement of professional identity. Respondents who completed training showed higher role satisfaction compared to those who did not (*p* = 0.0114). COVID-19 challenges included keeping up with emerging policies/procedures, CPs’ well-being, and inadequate funding to meet service needs; opportunities identified included service delivery expansion and CPs meeting community needs in a flexible manner. Respondents reported that sustainable payment models, expanding services, and geographic reach were important to the future of community paramedicine.

**Conclusions:**

Interprofessional collaboration is important to fulfill CPs roles. Role clarity and readiness could be improved, which aligns with the emerging nature of community paramedicine. The future of the community paramedicine care model is dependent on funding and expanding reach of services.

**Supplementary Information:**

The online version contains supplementary material available at 10.1186/s12913-023-09537-x.

## Background

Community paramedicine is an emerging field whereby community paramedics (CPs), emergency medical technicians (EMTs), or paramedics undergo additional training to work in expanded roles to deliver patient-centered care. Gaps in local community healthcare services drive the type of services delivered, which may include primary care, care coordination, and public health/preventive services [1–4]. Similarly, the training of CPs is guided by the program objectives and widely varies [4, 5]. A common requirement to practice as a CP is having a community paramedic education and at least 2 years of paramedic experience [4, 5]. Community paramedicine, which began in rural areas, is spreading globally with increasing adoption in non-rural settings [6, 7]. In the recent COVID-19 pandemic, CPs proactively engaged patients by conducting in-home assessments, identifying COVID-positive patients, and supporting self-isolated patients [8]. Evidence shows that CPs are the most common health providers utilized in community paramedicine/mobile integrated health (MIH) programs which are designed to address health gaps and reduce emergency resources utilization [5]. A nationwide survey across 129 community paramedicine/MIH programs in the U.S. reported that 9 of 10 programs utilize community paramedics [5]. Additionally, CPs represented 90% of the health personnel employed, followed by emergency medical technicians (EMTs) (29%), and case/social workers (24%) [5]. With these unique features, CPs are increasingly integrated with healthcare organizations to optimize patient outcomes, reduce or contain costs, and decrease nonurgent use of emergency resources [4, 9, 10].

CPs work collaboratively with health care providers, including physicians, nurses, and social workers, to tailor services to meet individual health needs [4]. Therefore, positive, mutual, and respectful relationship between CPs and other health care providers is essential to providing patient-centered care [11, 12]. However, transitioning from delivering urgent care services as an EMT to expanded roles in non-urgent services as a CP requires different skill sets. To provide non-urgent services, CPs must collaborate with other health care providers while considering patient needs and providers’ professional differences [13, 14]. This transition could impact how CPs perceive their professional identity. Understanding CPs’ professional identity is critical because it is the foundation for CPs' development of their professional role, interprofessional collaboration, and the way they interact with patients [15].

Moreover, CPs’ roles and responsibilities may be unclear to health providers and there is concern about overlapping roles [16]. Evidence indicates that role clarity (i.e., understanding of distinct professional roles) leads to a strong professional identity, while the opposite could lead to loss of professional identity [17]. As professional identity could impact interprofessional collaboration, and commitment to professional roles [18], it is essential to evaluate CPs’ perceptions of their transition from the provision of conventional urgent care services to non-urgent expanded roles. Hence, this study evaluated the viewpoint of CPs regarding their training, roles, role clarity, role readiness, role satisfaction, professional identity, and interprofessional collaboration.

Study objectives included:

1. To describe CPs’ demographic, training, roles, work, and program characteristics; COVID-19 pandemic experiences; and practice perceptions (role clarity, role readiness, role satisfaction, professional identity, interprofessional collaboration)
2. To evaluate the relationship between professional identity and role clarity.
3. To evaluate if professional identity and role satisfaction differ by training completion status.
4. To evaluate if the extent of interprofessional collaboration is associated with professional identity and if the extent of interprofessional collaboration differs by training completion and work experience.
5. To evaluate if the extent of interprofessional collaboration is associated with CPs’ roles.
6. To explore perceptions of the future of community paramedicine.

## Methods

### Study design, sampling, and distribution

A cross-sectional survey was conducted over 3 weeks in July/August 2020 using Qualtrics software. Potential study participants were identified using the National Association of Emergency Medical Technicians-mobile integrated health (NAEMT-MIH) listserv. The NAEMT consists of EMS professional members tasked with advocating for the provision of quality patient care [19]. Eligibility criteria were paramedics/EMTs who were ≥ 18 years old, currently practicing as community paramedics or a paramedic on a MIH team, and willing to participate in the study. An introduction letter containing the survey link was distributed via email. Using the NAEMT-MIH listserv of two digital email servers (MIH news/information and EMS leadership), an invitation letter containing the survey link was distributed by administrators of community paramedicine/MIH programs to 372 team members. Two follow-up reminder emails, in weekly increments, were also distributed. To enhance participation by respondents who met the eligibility criteria, two screening questions were included at the beginning of the survey: (1) *“Are you an emergency medical technician (EMT) or a paramedic?”* and (2) *“Are you actively working as a community paramedic or a paramedic on a MIH team?”* Participants that responded *‘yes’* to both questions advanced to complete the survey*.* Upon completion of the survey questionnaire, respondents were invited to enter a drawing for the chance to win an EMS medical gear. The study was approved by the University of Austin at Austin’s Institutional Review Board [Approval Number:2019–08-0052].

### Questionnaire

The development of the survey questionnaire was informed by the literature and by adapting previously validated instruments to align with CP practice [4, 5, 8, 20–22]. The questionnaire comprised 43 questions across ten sections: training characteristics, roles/COVID-19 pandemic experiences, practice perceptions (role clarity, role readiness, professional identity, role satisfaction, interprofessional collaboration), program characteristics, demographic/work characteristics, and future of community paramedicine.

Eleven questions assessed training characteristics: 1) training completion beyond on-the-job training (yes/no), 2) types of patient care training (yes/no), 3) types of interpersonal training (yes/no*)*, 4) duration of classroom/didactic training (none to 9 weeks or more), 5) duration of clinical training (none to 10 days or more), 6) classroom training mode (in-person, online, other), 7) clinical training mode (practice site, direct practice/experiential rotation, shadowing a clinician, other), 8) certification completion (yes/no*)*, 9) certification issuing agency (International Board of Specialty Certification, community college, local agency, other), 10) previous professional license (yes/no) and 11) type of license (Licensed Vocational or Practical Nurse (LVN, LPN), Registered Nurse (RN), Social Worker (LMSW, LCSW), other).

Fourteen questions assessed the type and frequency of roles (health assessment, medical procedures, disease management, medication management, medication administration, disease self-management, patient coordination, patient navigation, health education, health promotion, vaccine administration, injury prevention/safety assessment, urgent care, and other) performed by CPs in a typical week [4]. Using a 5-point Likert scale, responses were classified from 1 (Less than once a week) to 5 (Every day). To indicate roles not performed, a 0 (not applicable) option was included. Five questions assessed CPs’ experience during the COVID-19 pandemic: [8] 1) types of COVID-19 roles (in-home assessments of suspected or confirmed COVID-19 cases, identifying infected patients that require hospitalization, transporting infected patients for emergency visits, aiding self-isolated patients, and other roles,(yes/no for each role), 2) the impact of COVID-19 on CP roles (response on a 10-point Likert scale ranging from 0 = not at all to 10 = to a great extent), 3) personal protective equipment (PPE) access (Never, Rarely, Sometimes, Very Often, Always), 4) COVID-19 challenges (free text response), and 5) COVID-19 opportunities (free text response).

CP role clarity about professional roles, work objectives, and role expectations was assessed with a question consisting of 4-items [22]. Responses were captured using a 5-point Likert scale (1 = strongly disagree to 5 = strongly agree). The sum mean scores were calculated. Higher scores indicated a higher role clarity. A modified version of the professional identity of public health nurses (PIPN) questionnaire [20] was used to assess CPs’ professional identity. Three domains exist within the PIPN questionnaire: intention to develop professionally (4 items), confidence in own abilities (4 items), and occupational affinity (3 items). All items were scored using a 5-point Likert scale (1 = strongly disagree to 5 = strongly agree). Sum mean scores were calculated for each domain and overall items, with a higher score indicating a higher level of professional identity. To ensure consistency in patient care terminology used in the U.S., modifications were made to some words. For instance, the words ‘clients’ and ‘junior’ were replaced with ‘patients’ and ‘colleagues’, respectively.

The extent of interprofessional collaboration was evaluated using items from the Assessment of Interprofessional Team Collaboration Scale-II (AITCS-II) [21]. Three domains exist within the AITCS-II: partnership (8 items), cooperation (8 items), and coordination (7 items). For this study, 9 of 23 items (partnership [2 items] and cooperation [7 items]), were utilized in the study because the items aligned with CPs’ collaboration with other health professionals. Items from the coordination domain were not utilized because they assessed collective interprofessional collaboration. Items were scored on a 5-point Likert scale (1 = never to 5 = always). The sum mean scores were calculated for each domain and overall. A single question measured CPs viewpoint on the importance of interprofessional collaboration measured on a 10-point Likert scale (0 = not at all important to 10 = very important). A single question with a 5-point Likert scale measured role satisfaction (1 = very dissatisfied to 5 = very satisfied). CPs’ role readiness was assessed with a single item: *“From my first day as a community paramedic, I was adequately prepared to carry out my roles and responsibilities.”*A 5-point Likert scale measured response (1 = strongly disagree to 5 = strongly agree). Demographics (age, gender identity, race/ethnicity, educational level), work characteristics (CPs’ work hours, CPs work experience, previous EMT/paramedic experience), and program characteristics (practice setting, geographical region, CPs program duration, delivery model, patient population, funding, data sharing, outcomes documentation, and MIH practice) were measured using questions constructed by the research team and from evidence in the literature [4, 5]. Finally, CPs' perceptions of the future of community paramedicine were evaluated using two questions: *“Where do you see your local community paramedicine program in the next 3–5 years?”* and *“Where do you see the field of community paramedicine going in the next 10–20 years?”* The response was provided as free text.

To ensure the content validity of the questionnaire, two experts in community paramedicine evaluated each survey item for readability, interpretation, and content. Then the questionnaire was pilot tested by two CPs. Based on the feedback, minor modifications were made on five items to improve clarity and nine additional items were included in the final survey (see Supplement 1). The additional items were two eligibility screening questions and seven questions to evaluate the type of non-EMS license(s), previous EMT/paramedic experience, experiences during the COVID-19 pandemic, and the future of community paramedicine.

### Data analyses

Data analyses were conducted with SAS software (version 9.4, SAS Institute) and R package version 3.6.1. Cronbach’s alpha was used to measure the reliability of multi-item scales (role clarity, professional identity, and extent of interprofessional collaboration). Objective 1 was analyzed using descriptive statistics (means, standard deviations, frequencies, and ranges). To account for the non-parametric nature of the data, Spearman’s correlation and Wilcoxon Mann–Whitney U were used for objectives 2 and 3, respectively. Objective 4 was analyzed using Spearman’s correlation, Wilcoxon Mann–Whitney U, and Kruskal–Wallis tests. Finally, Objective 5 was analyzed with the Kruskal–Wallis test. The significance level was *p* < 0.05. However, the significance level was set at *p* < 0.01 for Objective 5 to reduce type 1 error inflation for multiple comparisons.

Qualitative content analyses explored free-text responses from Objective 1 (challenges/opportunities encountered during the COVID-19 pandemic) and Objective 6 (CPs’ perceptions on the future of community paramedicine). Responses obtained were evaluated independently and coded into categories by two members of the research team. To clarify any discrepancies, codes were re-examined independently by two other researchers.

## Results

The survey was distributed by program administrators to 372 participants that were either members of NAEMT or non-members who subscribed to NAEMT-MIH emails. However, only 111 participants responded to the survey. Of the 111 participants, 29 participants responded ‘no’ to either one or both screening questions. Thus, those participants were excluded because they were not EMTs or paramedics and actively practicing as community paramedics. Therefore, the total number of potentially eligible respondents that received the invitation was 343. Therefore, 82 participants responded ‘yes’ to both screening questions. Also, all 82 participants met the study criteria. Of these 82 respondents, 25 were excluded due to missing data. Of the 25 responses, 9 and 16 responses were excluded due to complete missingness (i.e., no response at all) and incomplete missingness (non-random missing data of ≥ 80%), respectively. Therefore, data analyses were conducted on responses from 57 participants after accounting for ineligibility and missingness. The response rate was 16.6% (57/343).

The characteristics of the study population are described below.

### Demographic and work characteristics and frequency of work roles

Table 1 provides a summary of the demographic and work characteristics, while Table 2 describes the type and frequency of CP roles performed by respondents. The mean age of the participants was 44.3 (standard deviation ( ±) 10.0) years. Participants were primarily men (80.4%) and non-Hispanic White (88.2%). The most common degree was a bachelor’s degree (26.9%). Participants, on average, worked 29.0 ± 15.8 h per week as CPs. Participants primarily had CP work experience of 4 years or more (30.8%) and had previously worked as EMTs or paramedics for 18.0 ± 9.9 years (Table 1). The most common role was ‘performing health assessments’ (96.5%) and the least common role performed was ‘vaccine administration’ (38.6%) (Table 2). Overall, the COVID-19 pandemic had a large impact on CPs’ roles (M = 7.6 ± 3.4/10 with 10 being high impact). ‘Conducting in-home assessments’ (64.8%) and ‘transporting COVID-19 infected patients’ (37.0%) were the most and least common COVID-19-related roles, respectively. Overall, 96.5% of participants had access to PPE, of which 76.4% reported that they ‘always’ had access to PPE.

**Table 1:** Community paramedic demographic and work characteristics (*N* = 52)

	**Mean** ± **SD [Range] or n (%)**
**Age (years) (*n* = 51)**	44.3 ± 10.0 [24.0 - 65.0]
**Gender Identity (*n* = 51)**	
Male	41 (80.4)
Female	10 (19.6)
**Race/ethnicity (*n* = 51)**^a^	
Non-Hispanic White	45 (88.2)
Hispanic or Latinx	4 (7.8)
American Indian or Alaska Native	1 (2.0)
Other^b^	2 (3.9)
**Educational Level (*n* = 52)**	
High school or GED	5 (9.6)
Technical college certificate	7 (13.5)
Associate degree	12 (23.1)
Bachelor’s degree	14 (26.9)
Master’s degree	10 (19.2)
Other^c^	4 (7.7)
**Work Hours (hours per week) (*n* = 47)**	29.0 ± 15.8 [3.0 - 48.0]
**Work Experience (years) (*n* = 52)**	
Less than 1 year	13 (25.0)
1 year to 2 years	8 (15.4)
3 years to 4 years	15 (28.8)
Greater than 4 years	16 (30.8)
**Previous EMT/Paramedic Experience (years) (*n* = 50)**	18.0 ± 9.9 [3.0 - 41.0]

*SD* Standard deviation, *GED* General education diploma, *EMT *Emergency medical technician

^a^Participants selected more than one response

^b^Other include human (*n* = 1), multi-cultural (*n* = 1)

^c^Other include doctorate (*n* = 2), US Navy Hyperbaric/Diving/Medic training (*n* = 1), Some college (*n* = 1)

± represents the standard deviation (SD)

**Table 2:** Type of community paramedic roles and how often each role was performed (*N* = 57)

	**N (%)**	**Everyday (n, %)**	**4 days (n, %)**	**2 to 3 days (n, %)**	**1 day (n, %)**	**Periodically (n, %)**	**NA (n, %)**
Perform health assessment	57 (100.0)	**32 (56.1)**	2 (3.5)	9 (15.8)	6 (10.5)	6 (10.5)	2 (3.5)
Perform medical procedures	57 (100.0)	8 (14.0)	2 (3.5)	7 (12.3)	5 (8.8)	9 (15.8)	**26 (45.6)**
Provide disease management	57 (100.0)	**26 (45.6)**	2 (3.5)	14 (24.6)	6 (10.5)	5 (8.8)	4 (7.0)
Perform medication management	57 (100.0)	**22 (38.6)**	3 (5.3)	12 (21.0)	10 (17.5)	6 (10.5)	4 (7.0)
Administer medications	57 (100.0)	11 (19.3)	2 (3.5)	**13 (22.8)**	8 (14.0)	11 (19.3)	12 (21.1)
Administer vaccines	57 (100.0)	0 (0.0)	0 (0.0)	1 (1.8)	4 (7.0)	17 (29.8)	**35 (61.4)**
Encourage patient to self-manage their conditions	56 (98.2)	**28 (50.0)**	2 (3.6)	12 (21.4)	4 (7.1)	6 (10.7)	4 (7.1)
Provide health education	57 (100.0)	**21 (36.8)**	4 (7.0)	7 (12.3)	6 (10.5)	12 (21.1)	7 (12.3)
Provide health promotion	57 (100.0)	10 (17.5)	1 (1.8)	11 (19.3)	4 (7.0)	13 (22.8)	**18 (31.6)**
Coordinate care	57 (100.0)	**22 (38.6)**	4 (7.0)	8 (14.0)	9 (15.8)	8 (14.0)	6 (10.5)
Navigate patients through the health care system	56 (98.2)	12 (21.4)	3 (5.4)	9 (16.1)	5 (8.9)	11 (19.6)	**16 (28.6)**
Perform injury prevention/safety assessment	56 (98.2)	**15 (26.8)**	5 (8.9)	10 (17.9)	8 (14.3)	9 (16.1)	9 (16.1)
Provide urgent care services	55 (100.0)	11 (20)	0 (0.0)	8 (14.6)	8 (14.6	**16 (29.1)**	12 (21.8)
Other^a^	29 (50.9)	3 (10.3)	0 (0.0)	0 (0.0)	0 (0.0)	3 (10.3)	**23 (79.3)**

Periodically = Less than a typical week i.e., rarely perform the role in a typical week; NA = Role not performed

Bolded numbers indicate the highest frequency of individual roles conducted in a typical week. The reverse is observed in the NA (role not performed) column

The frequency of performing roles was assessed by the number of days that CPs performed roles in a typical week based on most common roles identified in the primary literature

*NA* Not applicable (never performed or not an assigned responsibility)

^a^Other included COVID-19 education (every day, *n* = 1)

### Training characteristics and types of training

Tables 3 and 4 described CP training characteristics and types of community paramedicine training, respectively. Didactic and clinical training (Table 3) were primarily carried out using in-person mode (77.8%) and by shadowing a clinician (74.4%). The didactic training duration varied. The most commonly reported training duration was 9 weeks or more (21.7%) and 2 to 3 days (21.7%), while clinical training mostly took place across 10 days or more (50%). The most common types of patient care training were ‘identifying social needs affecting patient care’ (97.8%), and the least reported was ‘providing patient navigation’ (67.4%) (Table 4). However, interpersonal training was generally high (80.0% or higher) with ‘identifying socioeconomic factors’ (89.1%) as the most common interpersonal training. Upon completion of training, less than one-half of respondents (43.5%) were issued community paramedicine certification. Certification was mostly obtained from community colleges (35%) and local agencies (35%). Before becoming CPs, about one-fourth of the participants (28.1%) had professional licensure of some type (Table 3).

**Table 3:** Community paramedic training characteristics (*N* = 57)

	**n (%)**
**Training Completion (*n* = 57)**	
Yes	46 (80.7)
No	11 (19.3)
**Didactic/classroom Training (*n* = 46)**	
9 weeks or more	10 (21.7)
5 to 8 weeks	7 (15.2)
3 to 4 weeks	4 (8.7)
1 to 2 weeks	6 (13.0)
4 to 6 days	7 (15.2)
2 to 3 days	10 (21.7)
1 day or less	1 (2.2)
None	1 (2.2)
**Didactic Training Mode (*n* = 45)**^a^	
In-Person	35 (77.8)
Online (e.g., distance learning, webinar)	19 (42.2)
Other^b^	2 (4.4)
**Clinical Training (*n* = 46)**	
10 days or more	23 (50.0)
7 to 9 days	2 (4.4)
4 to 6 days	1 (2.2)
2 to 3 days	7 (15.2)
1 day or less	6 (13.0)
None	7 (15.2)
**Clinical Training Mode (*n* = 39)**^a^	
Rotation at a practice site	15 (38.5)
Direct practice/ experiential rotation	27 (69.2)
Shadowing a clinician	29 (74.4)
Other^c^	1 (2.6)
**Certification (*n* = 46)**	
Yes	20 (43.5)
No	26 (56.5)
**Certificate Issuing Agency (*n* = 20)**	
International Board of Specialty Certification	1 (5.0)
Community College	7 (35.0)
Local Agency^d^	7 (35.0)
Other^e^	5 (25)
**Previous Professional License (*n* = 57)**	
Yes	16 (28.1)
No	41 (71.9)
**Type of License (*n* = 16)**^a^	
Registered Nurse (RN)	3 (18.8)
Other^f^	14 (87.5)

^a^Participants selected more than one response

^b^Other included the University of Arkansas for Medical Sciences (*n* = 1); hospital job training (*n* = 1)

^c^Other included in-class (*n* = 1)

^d^Local agency included health agencies (United Health Care (*n* = 2), Center of Emergency Medicine of Western Pennsylvania (*n* = 1), Denver Health (*n* = 1), health system (*n* = 1)); state endorsement (*n* = 1)

^e^Other included EMS section of the Arkansas Department of Health (*n* = 1), Northwell Health System (*n* = 1), International Board of Specialty Certification (*n* = 1), state CP program (*n* = 1), university (*n* = 1)

^f^Other included paramedic (Board Certified Critical Care Paramedic (*n* = 1), Critical Care Emergency Medical Transport (*n* = 1), emergency medical technician (*n* = 2), Flight Paramedic Certification (*n* = 1), CP Certificate (*n* = 1)); Naturopathic Doctor (*n* = 1); community health worker (*n* = 1); athletic training (*n* = 1); spiritual mentor (*n* = 1)

**Table 4:** Types of community paramedics’ training (*N* = 46)

	**n**	**Yes** (n, %)	**No** (n, %)
**Patient Care**			
Perform disease-specific health assessment	46	43 (93.5)	3 (6.5)
Take patient’s medical history	46	44 (95.7)	2 (4.3)
Perform medical procedures	46	32 (69.6)	14 (30.4)
Provide chronic disease management	46	43 (93.5)	3 (6.5)
Administer/manage medications	45	39 (86.7)	6 (13.3)
Provide preventive care/education	46	39 (84.8)	7 (15.2)
Identify social needs affecting patient care (e.g., social characteristics, transportation)	46	45 (97.8)	1 (2.2)
Participate in community needs assessment/allocation of resources	46	39 (84.8)	7 (15.2)
Understand community paramedic’s roles	46	44 (95.7)	2 (4.3)
Perform safety assessment/injury prevention	46	42 (91.3)	4 (8.7)
Provide patient navigation	46	31 (67.4)	15 (32.6)
Serve as a patient advocate in the management of their health	46	40 (87.0)	6 (13.0)
Assess personal wellness	45	37 (82.2)	8 (17.8)
Other^a^	24	9 (37.5)	15 (62.5)
**Interpersonal**			
Therapeutic communication	46	39 (84.8)	7 (15.2)
Identification of socioeconomic factors	46	41 (89.1)	5 (10.9)
Patient health literacy	45	36 (80.0)	9 (20.0)
Interprofessional collaboration	46	39 (84.8)	7 (15.2)
Other^b^	27	9 (33.3)	18 (66.7)

^a^Other included social determinants of health (*n* = 2), readmission avoidance processes and hospital readmissions (*n* = 2), de-addiction from substance abuse (*n* = 1), hospice care (*n* = 1), out-patient detox, COVID testing and management (*n* = 1), all additional training provided by medical director (*n* = 1), self-trained – not included in curriculum (*n* = 1)

^b^Other included care coordination (*n* = 3); navigation (*n* = 1); incorporating family in visits and decision-making (*n* = 1); collaboration with external organizations, social services (foodbank) (*n* = 1); readmission (*n* = 1); on-the-job training – not included in curriculum (*n* = 1); self-trained—not included in curriculum (*n* = 1)

### Program characteristics

Table 5 describes program characteristics. Community paramedicine practice setting was most commonly located in metropolitan areas (77.4%). Programs were most commonly located in the Northeast region (41.5%). About 52.8% of respondents worked in programs that had been operating for 5 years or more. Health services were most commonly delivered in hospital-based settings (49.1%) with services most commonly provided to patients with chronic conditions (100.0%) and high EMS users (77.4%), while children (7.5%) were the least served. Participants most commonly reported funding by health care agencies (43.4%) and reported funding from the federal government least (5.7%). Data were commonly shared with collaborators by telephone (69.8%) and electronic patient record systems (67.9%). Health services utilization (71.7%) and patient-reported outcomes (62.2%) such as health-related quality of life and patient satisfaction, were the most commonly documented outcomes. The types of outcomes documented by the programs were unknown by 20.8% of participants. Sixty percent of respondents were practicing in MIH programs.

**Table 5:** Community paramedicine program characteristics (*N* = 53)

	**n (%)**
**Practice Setting (*n* = 53)**	
*Non-metropolitan Setting*	*12 (22.6)*
Small rural (less than 10,000 residents)	2 (3.8)
Large rural (10,000 to 49,999 residents)	10 (18.9)
*Metropolitan Setting*	*41 (77.4)*
Small Metro (Less than 250,000 residents)	14 (26.4)
Medium Metro (250, 000 to 999,999 residents)	11 (20.8)
Large Metro (1 million or more residents)	16 (30.2)
**Geographical Region (*n* = 53)**	
Northeast	22 (41.5)
Midwest	7 (13.2)
South	8 (15.1)
West	16 (30.2)
**Program Duration (*n* = 53)**	
Less than 1 year	10 (18.9)
1 to 2 years	5 (9.4)
3 to 4 years	10 (18.9)
5 years or more	28 (52.8)
**Delivery Model (*n* = 53)**^a^	
Fire department	10 (18.9)
Hospital-based	26 (49.1)
Public – county	8 (15.1)
Public – city	1 (1.9)
Public – regional	2 (3.8)
Public utility model	3 (5.7)
Private (for-profit)	10 (18.9)
Private (nonprofit)	8 (15.1)
Law enforcement	1 (1.9)
Other^b^	2 (3.8)
**Patient Population (*n* = 53)**^a^	
Individuals with chronic conditions	53 (100.0)
Individuals with a disability	35 (66.0)
Homeless individuals	22 (41.5)
Individuals with mental health conditions	33 (62.3)
Individuals with substance/alcohol abuse	31 (58.5)
Uninsured individuals	24 (45.3)
High EMS users	41 (77.4)
High ED users	37 (69.8)
Individuals in hospice care	18 (34.0)
Older adults (≥ 65 years)	36 (67.9)
Children	4 (7.5)
Other^c^	5 (9.4)
**Funding (*n* = 53)**^a^	
Foundation/charitable grants	12 (22.6)
Federal government	3 (5.7)
State government	6 (11.3)
Local government	7 (13.2)
Insurance providers	10 (18.9)
EMS departments	10 (18.9)
Health care agencies	23 (43.4)
Don’t know	5 (9.4)
Other^d^	5 (9.4)
**Data Sharing (*n* = 53)**^a^	
Electronic patient record systems	36 (67.9)
Health information exchange systems	13 (24.5)
Encrypted email	27 (50.9)
Faxing	13 (24.5)
Telephone	37 (69.8)
Manually (pen and paper)	1 (1.9)
Other^e^	6 (11.3)
**Outcomes Documentation (*n* = 53)**^a^	
Health services utilization (e.g., hospital readmission/ admissions)	38 (71.7)
Cost savings	21 (39.6)
Patient clinical outcomes (e.g., blood pressure and blood glucose control)	30 (56.6)
Patient-reported outcomes (e.g., patient satisfaction, health-related quality of life)	33 (62.2)
Process measures (e.g., referrals, immunizations)	22 (41.5)
Other^f^	1 (1.9)
Don’t know	11 (20.8)
**MIH Practice (*n* = 53)**	
Yes	32 (60.4)
No	31 (39.6)
**MIH Team Operations (*n* = 31)**	
Independent (I work by myself in collaboration with medical oversight)	12 (38.7)
Pre-hospital (I work with another paramedic or an EMT)	9 (29.0)
Integrated (I work with another health care professional e.g., physician, nurse, social worker)	9 (29.0)
Other^g^	1 (3.2)

*ED* Emergency department, *EMS* Emergency medical services, *EMT* Emergency medical technician, *MIH* Mobile integrated health

^a^Participants selected more than one response

^b^Other included insurance agency (*n* = 1); accountable care organizations (*n* = 1); independent local government (*n* = 1)

^c^Other included acute/sub-acute patients enrolled in home care (*n* = 1); post-discharged patients (*n* = 1); patients with high-stakes surgery (*n* = 1); veterans (*n* = 1); patients across all age groups (*n* = 1)

^d^Other included CP program budget (*n* = 2); tax (*n* = 1); variety of revenue streams (*n* = 1); not funded (*n* = 1)

^e^Other included telehealth (*n* = 4); in-person (*n* = 1)

^f^Other included insurance program scorecards (*n* = 1)

^g^Other included fire service (*n* = 1)

### Practice perceptions

Table 6 describes study participants' practice perceptions. Role clarity, professional identity, role satisfaction, and the extent of interprofessional collaboration highly varied, with mean scores of 15.5 ± 4.3 (range 4.0–20.0), 46.8 ± 6.1 (range 30.0–55.0), 4.4 ± 0.9 (range 1.0–5.0), and 40.1 ± 4.2 (range 25.0–45.0), respectively. However, role readiness was neutral with mean scores of 3.3 ± 0.8 (range 2.0–4.0). Despite this, the perception of the importance of interprofessional collaboration was high with a mean score of 9.5 ± 0.9 (range 7.0–10.0). Participants primarily collaborated with physicians (94.3%). The reliability of the multi-item scales was high with Cronbach’s alpha values ranging from 0.83 to 0.90.

**Table 6:** Community paramedics’ practice perceptions (*N* = 54)

	**Mean** ± **SD [Range] or n (%)**
**Role Clarity (*n* = 54)**	15.5 ± 4.3 [4.0 - 20.0]
**Role Readiness (*n* = 43)**	3.3 ± 0.8 [2.0 - 4.0]
**Role Satisfaction (*n* = 53)**	4.4 ± 0.9 [1.0 - 5.0]
**Professional Identity (*n* = 53)**	46.8 ± 6.1 [30.0 - 55.0]
Professional development	16.0 ± 2.9 [8.0 - 20.0]
Confidence in roles	17.0 ± 2.3 [8.0 - 20.0]
Professional pride	13.8 ± 1.8 [7.0 - 15.0]
**Types of Interprofessional Collaboration (*N* = 53)**	
Physicians	50 (94.3)
Nurse practitioners	40 (75.5)
Physician assistants	35 (66.0)
Pharmacists	29 (54.7)
Registered nurses	47 (88.7)
Licensed vocational nurses	17 (32.1)
Social workers	44 (83.0)
Other^a^	16 (30.2)
**The extent of Interprofessional Collaboration (*N* = 53)**	40.1 ± 4.2 [25.0 - 45.0]
Partnership	8.2 ± 1.6 [5.0 - 10.0]
Cooperation	31.9 ± 3.1 [19.0 - 35.0]
**Importance of Interprofessional Collaboration (*N* = 53)**	9.5 ± 0.9 [7.0 - 10.0]

^a^Other included health agencies/providers (health care navigators, health system administrators, caseworkers/managers, patient care aides, nutritionist, radiology technicians, occupational therapist, physiotherapist, dentistry, optometry, mental health professionals, wound clinics, town/city health departments, department of human services) (*n* = 13); social service agencies (Food/Nutrition assistance program, food pantries, housing assistance services, transportation services, area churches, free clinics, housing police) (*n* = 2); home agencies (group home staff, nursing homes, home health agencies) (*n* = 2); other community paramedics and allied health providers (*n* = 2); crisis intervention team (*n* = 1); American Automobile Association (*n* = 1); law enforcement (*n* = 1); county workers (*n* = 1)

± represents the standard deviation (SD)

The results from the inferential analyses are discussed below.

### Professional identity, role clarity, and extent of interprofessional collaboration

Results for inferential analyses showed that higher levels of role clarity (rho = 0.0013, *p* = 0.0013) and extent of interprofessional collaboration (rho = 0.4, *p* = 0.0015) significantly enhanced professional identity.

### Role satisfaction, professional identity, and extent of interprofessional collaboration by training completion status

There was a significant difference in the mean rank scores of role satisfaction between participants that completed training and those that did not (U = 29.4 vs 16.7, *p* = 0.0114). However, no significant difference was observed in the mean rank scores of professional identity (U = 28.9 vs 18.7, *p* = 0.0657) and the extent of interprofessional collaboration (U = 27.8 vs. 23.5, *p* = 0.4326) by training completion status.

### Extent of interprofessional collaboration and work experience

Among the work experience groups, the mean rank scores of the extent of interprofessional collaboration in at least one work experience group was statistically significantly different from the other work experience groups (X^2^ = 8.5, *p* = 0.0374). However, pairwise two-multiple comparisons of interprofessional collaboration across work experience groups showed no significant difference in the extent of interprofessional collaboration across the paired work experience groups.

### Extent of interprofessional collaboration and CPs’ roles

Participants who performed patient navigation (X^2^ = 12.1, *p* = 0.0023), health promotion (X^2^ = 11.2, *p* = 0.0037), and injury prevention/safety assessments (X^2^ = 18.6, *p* < 0.0001) roles showed significant differences in the extent of interprofessional collaboration mean rank scores. Also, CPs who performed roles at least one day per week reported greater interprofessional collaboration compared to participants who did not perform the respective roles [patient navigation (33.7 vs 18.5, *p* = 0.0071), health promotion (34.1 vs 21.3, *p* = 0.0070), and injury prevention/safety assessments (32.8 vs 8.5, *p* = 0.0001)]. However, there was no significant association between the extent of interprofessional collaboration and the other role types. The content analysis of open-ended responses revealed the following findings:

### COVID-19 pandemic experiences

#### COVID-19 challenges

Participants reported that it was challenging to constantly tailor services to align with changing COVID-19 policies/procedures, a decline in service delivery, negative impacts on the well-being of CPs, and inadequate funding to meet service needs. Further, participants expressed that it was difficult to tailor visits/services to recommended COVID-19 guidelines. Also, they experienced a decline in PPE supplies, and difficulty in donning PPE gear. Additionally, due to the COVID-19 pandemic, some programs were either temporarily shut down or suspended leading to inconsistent service delivery. Difficulties in health assessments (e.g., medical testing and tracking), and patient navigation (e.g., assisting patients procure health self-assessment tools) roles were also expressed. CPs’ well-being was also affected by both emotional (e.g., increased stress, anger, discouragement, and difficulty in maintaining a positive attitude) and occupational (fear of infection, concern for family safety) negatives highlighted due to the COVID-19 pandemic.

#### COVID-19 opportunities

The COVID-19 pandemic provided opportunities to expand service delivery especially because CPs could be flexible in meeting community needs. Participants provided health services care at convenient and accessible locations (e.g., in-home care), identified patients that required hospitalization, engaged in treatment and referral activities, and mitigated patient despair/anxiety, thus fostering strong positive relationships with patients. Telehealth services provided enhanced avenues to regularly communicate with patients which contributed to building relationships with patients. Program growth and expansion were reported as new programs were established to meet the constantly evolving health needs. Also, participants experienced enhanced partnership and program expansion with insurance agencies including private payers.

### Perceptions on the future of community paramedicine

Participants envisioned sustainable payment models (e.g., routine MIH-coded billing mechanisms, the Emergency, Triage, Treat, and Transport (ET3) model, and adequate compensation by payers), a transformation of community paramedicine as the standard of EMS care, service delivery and reach expansion, increased telehealth utilization, and the implementation of educational standards were important to the future of community paramedicine. Participants envisioned expansions in geographical locations, practice, and collaboration, coupled with enhanced adoption of CPs services by various partners (e.g., EMS, fire-based EMS, and the public health sector). Enhancement of CPs education/training curriculum such as additional certifications (e.g., master’s degree), and specialties (e.g., pediatricians, behavioral health) was recommended.

## Discussion

In our study, participants reported that CPs are well-positioned to meet community health needs by conducting a wide variety of services tailored to evolving health needs. These findings align with those in previous studies highlighting CPs providing health care services across various settings and disease states [4, 5, 23, 24]. Similar to other studies [24, 25], the most common outcomes reported were health services utilization (71.7%) followed by patient-reported outcomes (62.2%). However, 20% of the respondents were not aware of which outcomes were being documented. This could reflect a lack of communication between organizations and individual CPs or undefined processes for documentation in emerging programs. Furthermore, this could impact the quality of documentation and presents an opportunity to educate CPs on the relevance of documentation of key program outcomes indicators to improve health outcomes and achieve program objectives. As suggested by Chan et al., 2019, outcomes reporting is likely necessary to demonstrate value to health agencies and payers [24]. To address gaps in knowledge about outcomes and documentation, programs may need to provide education and training to CPs who are in the field about key outcome indicators and documentation processes. The Mobile integrated health-community paramedicine (MIH-CP) Outcomes Measures Projects initiated by a group of researchers and leaders in MIH-CP programs provide a uniform set of comprehensive outcome measures for MIH-CP programs [26]. This could be a valuable document to inform emerging programs on potential program outcomes and goals. Also, improved interoperability of data-sharing systems could be an avenue to a more patient-centric approach by unifying data to assess health outcomes and measure performance in service delivery.

CP roles, as expected, were focused on primary care (such as ‘perform health assessments’ (96.5%), ‘provide disease management’ (93.0%), ‘perform medication management’ (93.0%), and ‘encourage self-management of health conditions’ (92.9%)). Among the public health and preventive roles (e.g., health education (87.7%), health promotion (68.4%), vaccine administration), vaccine administration was performed the least (38.6%). Although vaccine administration was the least reported role performed, evidence highlights that CPs participate in community-wide immunization programs, especially in rural settings. Therefore, utilizing CPs for massive vaccination programs could be an area for further service expansion, particularly in rural settings and public health emergencies [27, 28]. Therefore, future studies may be required to evaluate potential reasons for minimal vaccination and how CPs’ training could be structured to improve vaccination.

A similar trend was observed for COVID-19-related roles with respondents conducting primary care roles such as ‘conducting in-home assessments’ (64.8%), ‘identifying infected patients that require hospitalization’ (59.2%), ‘supporting self-isolated patients’ (57.4%), and ‘transporting infected patients’ (37.0%). The study findings show that participants were flexible to adapt to the evolving COVID environment to meet the community's needs, despite the challenges of COVID-19. Therefore, CPs engaged with patients and provided patient-centric care at convenient places and connected them to health providers as seen in previous studies [2, 29, 30]. However, challenges encountered with COVID-19 such as the negative impact on CPs’ well-being, highlight the need for CPs to keep up with emerging public health policies and to be prepared to proactively deliver and effectively respond to future public health crises. Therefore, training tailored to public health crisis response could be necessary. Also, favorable work conditions (e.g., adequate staffing, sustainable reimbursements) and resources for public health emergency preparedness should be incorporated.

CP training commonly included topics on patient care, socioeconomic factors affecting patients’ health, and interprofessional collaboration. Socio-determinant of health contributions to health disparities is gaining wide attention on its role in health disparities, therefore, the EMS Agenda 2050 calls for greater integration of social determinants of health in CP training and education programs [31]. However, irrespective of training, role readiness was on average, neutral, among participants with a mean score of 3.3/5.0. Training based on program goals could result in honing skill sets in relevant areas [4]. For CPs to be sufficiently equipped for their roles, a more standardized curriculum structured to include core competencies with the flexibility for programs to customize portions of the curriculum to meet local health care needs may be required [24, 25, 32, 33]. The training curriculum could be enhanced by including additional topics to adequately prepare CPs for their roles. For instance, incorporating public health emergency response training will prepare CPs for future public health crises, while role clarity training could enhance professional identity which will be beneficial in interprofessional settings [4]. With the focus on value-based care with payment tied to the provision of quality care and improved health outcomes, an opportunity exists to increase the utilization of telehealth services to improve health service delivery especially in resource-constrained circumstances and during public health emergency responses [34].

Study findings showed respondents’ viewpoints varied in role clarity, professional identity, and role satisfaction. CPs performing diverse health care roles based on varying program goals could lead to differences in patient interaction and interprofessional collaboration which could explain these variations [4, 35–37]. Positive relationships of professional identity with role clarity (*p* = 0.0013) and interprofessional collaboration (*p*= 0.0015) demonstrate that professional identity could be improved by having clearly defined roles and working with multidisciplinary teams to render health services [17, 36]. Overall, training improved role satisfaction with significantly higher role satisfaction among participants that completed training (of any kind), highlighting the need for CPs’ education/training curriculum to be standardized to enhance role satisfaction. Supporting this, studies among nurses show that improving role satisfaction enhanced job performance and improved the quality-of-care delivery [38, 39]. Therefore, training should be adequately structured to equip CPs to address health needs, while enhancing role satisfaction and improved quality of care. Training could be structured in an interprofessional setting to promote interaction and enhance the learning of interprofessional collaboration skills with other health providers. Also, continual interprofessional development activities post-training will continuously foster collaboration among providers.

CPs collaborate with various types of health care providers with physicians (94.3%), registered nurses (88.7%), and social workers (83.0%)) being the most common collaborators [4, 24]. Participants rated interprofessional collaboration as very important (mean score of 9.5 ± 0.9) in the delivery of their services. This is expected as interprofessional collaboration skills are essential for effective interaction between CPs and other health providers to continuously meet the evolving needs of patients [4, 40].

Given that non-sustainable funding was a primary concern for programs, sustainable reimbursement models (such as fee-for-service, MIH-coded billing, ET3 payment models) should be designed and implemented to improve the sustainability of community paramedicine [23]. Recently, the ET3 fee-for-service payment model, a 5-year payment model providing post-hospital care for Medicare beneficiaries and state Medicaid agencies was initiated by the Centers for Medicare and Medicaid Services (CMS) [41, 42]. This is a promising initiative, therefore, future research is necessary to evaluate if the adoption of the ET3 approach could be a sustainable option.

The EMS Agenda 2050 proposed a patient-centric EMS system that will be reliable in addressing rising and unpredictable health needs, employ integrated data sharing systems and telehealth systems, incorporate sustainable payment models, address sociocultural needs, document and track metrics, foster interprofessional collaboration, improve coordination of care, and continually provide value-based services [31]. Findings from the current study align with the goals of EMS Agenda 2050 of providing value by addressing health and sociocultural needs, engaging in interprofessional collaboration and care coordination, reporting relevant health outcomes, and developing sustainable payment models.

This study is not without limitations. Firstly, the study was conducted using a cross-sectional design with responses captured at a single point in time. Secondly, the sample size was small. This could lead to wide variability in responses and could impact the study findings which may lead to bias. Since the study was conducted during the COVID-19 pandemic, CPs were actively busy with patient care and may not have responded to the survey. However, the perceptions of the study respondents may reflect those of the CPs in their respective programs and geographical settings. Thirdly, the survey and follow-up reminder emails were distributed by community paramedicine and MIH program administrators rather than the research team, and this may have contributed to the low response rate. Furthermore, the administration of the survey during the pandemic may have resulted in lower responses as the work burden during this time was high. Fourthly, participants were recruited using convenience sampling, therefore, there is potential for biases in the study. For instance, selection bias could be present as only interested CPs may have participated in the study. However, responses were captured nationally and across geographical settings. Recall bias could also be an issue as some survey items required responses from past details or events. Fifthly, the response categories provided for CPs’ work experience and program duration may not be adequate to capture the variety of experience and could result in misclassification errors. Finally, the study findings may not be representative of female CPs as respondents were primarily males (80.4%). Future studies to assess female CPs’ perceptions may be required. However, as the study was a national study, findings could be relevant to a variety of geographical and practice settings.

## Conclusion

CPs are consistently meeting the evolving health needs of the community. To continue advancing CP roles, additional training on role clarity, readiness, professional identity, and interprofessional collaboration is needed. To improve professional identity, roles should be clearly defined for effective role implementation and the advancement of the community paramedicine care model. For the sustainability of the community paramedicine care model, consistent reimbursement models, a transition to community paramedicine as the standard of care for EMS, and service delivery and geographic reach expansions are vital. Enhanced partnerships, utilization of telehealth systems during public health emergencies, improved standardization of training curriculum, development of sustainable reimbursement models, and proactive public health crisis preparedness should be prioritized with the evolution of the community paramedicine care model.

## Supplementary Information

**Additional file 1: Supplement 1.** Web-based survey questionnaire.

## Data Availability

Upon a reasonable request, the data and code for data analysis will be made available by the corresponding author.

## Abbreviations

AITCS-II: Assessment of Interprofessional Team Collaboration Scale-II
CMS: Centers for Medicare and Medicaid Services
CPs: Community Paramedic
EMS: Emergency medical services
EMTs: Emergency medical technicians
ET3: Emergency, Triage, Treat, and Transport
LCSW: Licensed Clinical Social Worker
LMSW: Licensed Master of Social Work
LVN: Licensed Vocational Nurse
LPN: Licensed Practical Nurse
NAEMT: National Association of Emergency Medical Technicians
MIH: Mobile integrated health
MIH-CP: Mobile integrated health-community paramedicine
PPE: Personal protective equipment
RN: Registered nurse
