# Supplementary material for: An examination of the emerging field of community paramedicine: a national cross-sectional survey of community paramedics
Source: BMC Health Serv Res. 2023 May 23;23:516. doi: 10.1186/s12913-023-09537-x (PMC10204027; doi:10.1186/s12913-023-09537-x)
Supplement: Supplementary file 1 — Additional file 1: Supplement 1. Web-based survey questionnaire. [file 12913_2023_9537_MOESM1_ESM.docx]

**Supplement 1: Web-Based Survey Questionnaire Script**

Welcome! Your participation is appreciated.

This survey consists of 10 sections about your community paramedicine training, roles, role clarity, readiness for roles, professional identity, role satisfaction, and interprofessional collaboration. We will also assess your opinion on the future of community paramedicine.

**SCREENING QUESTIONS**

1. **Are you an emergency medical technician (EMT) or a paramedic?**

 Yes

 No

1. **Are you actively working as a community paramedic or as a paramedic on a mobile integrated health (MIH) team?**

 Yes

 No

*(For “No” responses in either question 1 or 2, the survey will automatically be set to end as participants are not eligible for the study).*

**SECTION 1: COMMUNITY PARAMEDIC TRAINING**

*This set of questions will help us understand the type and extent of your training in community paramedicine.*

***Instruction:*** *Please select the option that best aligns with your community paramedic training.*

**Have you completed additional training beyond on-the-job training to prepare you for your role as a community paramedic?**

 Yes

 No

*(For “No” response in question 1, the survey will automatically be skipped to question 10).*

1. **Please indicate yes or no for each type of patient care training you completed to prepare you for your role as a community paramedic.**

| **a. Perform disease-specific health assessment**  (e.g., physical, and mental health assessment, quality of life, health risk assessment) | Yes   | No   |
| --- | --- | --- |
| **b. Take patient’s medical history**  (e.g., perform comprehensive patient history and documentation) | Yes   | No   |
| **c. Perform medical procedures**  (e.g., wound care and sepsis management, in-home infusion, airway maintenance, urinary catheterization and maintenance, peripheral intravenous access maintenance) | Yes   | Yes   |
| **d. Provide chronic disease management**  (e.g., patient education, in-home care, preventive care, care plans  interpretation, point-of-care testing such as blood pressure monitoring,  complete blood count, blood/fluid chemistry profile, metabolic profile) | Yes   | No   |
| **e. Administer/manage medications**  (e.g., medication monitoring, reconciliation, adherence, management of adverse drug reactions) | Yes   | No   |
| **f. Provide preventive care/education**  (e.g., screen for chronic diseases, provide education for chronic diseases and community resources, oral health education and screening) | Yes   | No   |
| **g. Identify social needs affecting patient care**  (e.g., social characteristics, transportation) | Yes   | No   |
| **h. Participate in community needs assessment/allocation of resources**  (e.g., social services, housing, mental care) | Yes   | No   |
| **i. Understand community paramedic’s roles**  (e.g., roles in primary care, public health, and the health care systems) | Yes   | No   |
| **j. Perform safety assessment/injury prevention**  (e.g., fall/injury prevention and safety protocol) | Yes   | No   |
| **k. Provide patient navigation**  (e.g., guiding patients through and around barriers in the complex health  care system) | Yes   | No   |
| **k. Provide patient navigation**  (e.g., guiding patients through and around barriers in the complex health care system) | Yes   | No   |
| **l. Serve as a patient advocate in management of their health**  (e.g., provide patient support in decision making, protect patient privacy and confidentiality) | Yes   | No   |
| **m. Assess personal wellness**  (e.g., warning signs of stress, stress management, stages of grief) | Yes   | No   |
| **n. Other (please specify)** ____ | Yes   | No   |

1. **Please indicate yes or no for each type of interpersonal training you completed to prepare you for your role as a community paramedic.**

| **a. Engage patients in therapeutic communication**  (e.g., counseling, and motivational interviewing such as reflective listening skills, give affirmations) | Yes   | No   |
| --- | --- | --- |
| **b. Identify cultural factors affecting patient care**  (e.g., manage cultural differences such as language, religion, race, sexual orientation) | Yes   | No   |
| **c. Improve patient health literacy**  (e.g., empower patients with health information to make more informed decisions) | Yes   | No   |
| **d. Participate in interprofessional collaboration** | Yes   | No   |
| **e. Other (please specify)** ____ | Yes   | No   |

1. **How much didactic/classroom training did you receive for your role as a community**

**paramedic**?

 None

 1 day or less

 2 to 3 days

 4 to 6 days

 1 to 2 weeks

 3 to 4 weeks

 5 to 8 weeks

 9 weeks or more

*(For “None” response in question 4, the survey will automatically skip to question 6)*

**5. How was the didactic/classroom training delivered? (select all that apply)**

 In-person

 Online (e.g., distance learning, webinar)

 Other (please specify) ____

**6. How much clinical training did you receive for your role as a community paramedic**?

 None

 1 day or less

 2 to 3 days

 4 to 6 days

 7 to 9 days

 10 days or more

*(For “None” response in question 6, the survey will automatically skip to question 8).*

1. **How was the clinical training delivered? (select all that apply)**

 Rotation at a practice site

 Direct practice/experiential rotation

 Shadowing a clinician

 Other (please specify) ___

1. **Did you obtain a community paramedicine certification upon completion of the community paramedicine training program?**

 Yes

 No

*(For “No” response in question 8, the survey will automatically skip to question 10).*

1. **What type of agency/organization issued the community paramedicine certificate?**

 International Board of Specialty Certification

 Community college

 Local program (please specify) ____

 Other (please specify) ____

1. **Have you obtained any other professional license(s)?**

 Yes

 No

*(For “No” response in question 10, the survey will automatically skip to Section 2).*

1. **Which license(s) have you obtained?**

 Licensed Vocational or Practical Nurse (LVN, LPN)

 Registered Nurse (RN)

 Social Worker (LMSW, LCSW)

 Other (please specify) ____

**SECTION 2: COMMUNITY PARAMEDIC ROLES**

*The table below lists the most common community paramedic roles identified in the primary literature.*

***Instructions:*** *Please consider your current roles/responsibilities as a community paramedic and select the option which is most relevant.*

1. **In your current role, in a typical week, how frequently do you do the following activities?**

*If you never perform the task or the task is not a role/responsibility, please check the ‘not applicable (NA)’ option.*

|  | **Everyday** | **4 days** | **2 to 3 days** | **1 day** | **Periodically (Less than a typical week)** | **NA** |
| --- | --- | --- | --- | --- | --- | --- |
| **a. Perform health assessment**  (e.g., medical history, physical and mental health assessment, health risk assessment, health screening, quality of life assessment) |  |  |  |  |  |  |
| **b. Perform medical procedures**  (e.g., transfusions, urinary catheterization, suturing, feeding tube insertion) |  |  |  |  |  |  |
| **c. Provide disease management**  (e.g., identify and treat disease-related symptoms, disease-specific education, in-home care, post-discharge care, point-of-care testing such as blood glucose test) |  |  |  |  |  |  |
| **d. Perform medication management**  (e.g., educate patients on medications, medication reconciliation, adherence, and adherence verification) |  |  |  |  |  |  |
| **e. Administer medications** |  |  |  |  |  |  |
| **f. Administer vaccines**  (e.g., pneumococcal and flu vaccines) |  |  |  |  |  |  |
| **g. Encourage patient to self-manage their conditions**  (e.g., self-monitoring of health parameters (blood pressure, blood glucose), development of individualized health plans, patient education, lifestyle modification instruction, wound-care instructions) |  |  |  |  |  |  |
| **h. Provide health education**  (e.g., nutrition education, family safety, and poison control information) |  |  |  |  |  |  |
| **i. Provide health promotion**  (e.g., first aid training, public education programs on healthy lifestyles, death and illness prevention) |  |  |  |  |  |  |

|  | **Everyday** | **4 days** | **2 to 3 days** | **1 day** | **Periodically (Less than a typical week)** | **NA** |
| --- | --- | --- | --- | --- | --- | --- |
| **j. Coordinate care**  (e.g., coordination of care with other health care team members, referral to community resources and health providers, electronic medical record charting) |  |  |  |  |  |  |
| **k. Navigate patients through the health care system**  (e.g., transportation, assistance with finances (advising, disability payments, health insurance), obtaining medications and medical devices) |  |  |  |  |  |  |
| **l. Perform injury prevention/safety assessment**  (e.g., Home safety assessment, fall risk assessment, injury prevention education) |  |  |  |  |  |  |
| **m. Provide urgent care services**  (e.g., transportation to the emergency department determined by evaluation, acute patient assessment) |  |  |  |  |  |  |
| **n. Other (please specify)** ____ |  |  |  |  |  |  |

1. **On a scale of 0 to 10, to what extent did the coronavirus (C0VID-19) pandemic impact your roles/responsibilities as a community paramedic?**

1 2 3 4 5 6 7 8 9 10

Not at all (0) To a great extent (10)

1. **Please check yes or no to indicate whether or not you perform any of the COVID-19 roles listed below.**

|  | Yes | No |
| --- | --- | --- |
| 1. Conduct in-home assessments for patients with suspected or confirmed COVID-19 |  |  |
| 1. Identify infected patients that require hospitalization |  |  |
| 1. Transport infected patients to emergency departments |  |  |
| 1. Support self-isolated patients |  |  |
| 1. Other (please specify) ____ |  |  |

1. **During the COVID-19 pandemic, how often have you had access to personal protective equipment (PPE) recommended by evidence-based guidelines?**

 Never

 Rarely

 Sometimes

 Very Often

 Always

1. **What has been the most significant challenge you have encountered during the COVID-19 pandemic? (Please specify)**
2. **What has been the most significant opportunity you have encountered during the COVID-19 pandemic? (Please specify)**

**SECTION 3: ROLE CLARITY**

*The following questions will assess the clarity of your professional role as a community paramedic.*

1. **Please select the option that best corresponds to your level of agreement for each question.**

|  | **Strongly Disagree** | **Disagree** | **Neutral** | **Agree** | **Strongly Agree** |
| --- | --- | --- | --- | --- | --- |
| 1. I am clear about my professional roles/responsibilities. |  |  |  |  |  |
| 1. My work objectives are well defined. |  |  |  |  |  |
| 1. I am clear about what other health professionals expect of me. |  |  |  |  |  |
| 1. I am clear about what patients expect of me. |  |  |  |  |  |

**SECTION 4: ROLE READINESS**

*This section will assess your readiness for professional roles.*

*Please select the option that best corresponds to your level of agreement.*

1. **From my first day as a community paramedic, I was adequately prepared to carry out my roles and responsibilities.**

 Strongly Agree

 Agree

 Neutral

 Disagree

 Strongly Disagree

**SECTION 5: PROFESSIONAL IDENTITY**

*The following questions are about your perceptions of your professional identity as a community paramedic.*

1. **Please select the option that best corresponds to your level of agreement for each question.**

|  | **Strongly Disagree** | **Disagree** | **Neutral** | **Agree** | **Strongly Agree** |
| --- | --- | --- | --- | --- | --- |
| 1. I have goals for developing as a community paramedic. |  |  |  |  |  |
| 1. I want to develop further as a community paramedic. |  |  |  |  |  |
| 1. I make self-driven efforts to develop as a community paramedic. |  |  |  |  |  |
| 1. I have a role model who is a community paramedic. |  |  |  |  |  |
| 1. I have confidence in my abilities as a community paramedic. |  |  |  |  |  |
| 1. My experience is useful to my colleagues. |  |  |  |  |  |
| 1. I can incorporate the needs of patients and organizations to achieve administrative goals. |  |  |  |  |  |
| 1. I am depended on by my patients. |  |  |  |  |  |
| 1. I feel pride in working as a community paramedic. |  |  |  |  |  |
| 1. I think that a community paramedic’s work is interesting. |  |  |  |  |  |
| 1. I feel that community paramedics have unique abilities. |  |  |  |  |  |

**SECTION 6: ROLE SATISFACTION**

*This question will assess your satisfaction with your professional role as a community paramedic.*

*Please select the option that best corresponds to your level of satisfaction.*

1. **Overall, how satisfied, or dissatisfied are you with your role as a community paramedic?**

 Very Dissatisfied

 Dissatisfied

 Neutral

 Satisfied

 Very Satisfied

**SECTION 7: INTERPROFESSIONAL COLLABORATION**

*The section contains questions about your collaboration with other health professionals as a community paramedic.*

1. **What type of health professionals have you worked with as a community paramedic? (select all that apply)**

 Physicians

 Nurse Practitioners

 Physician Assistants

 Pharmacists

 Registered Nurses

 Licensed Vocational Nurses

 Social Workers

 Other (please specify) ______

1. **On a scale of 1 to 10, please indicate the importance of collaboration with other health care professionals in your role as a community paramedic.**

1 2 3 4 5 6 7 8 9 10

Not at all Very

important (0) important (10)

**3. When working with other health professionals, I:**

|  | **Never** | **Rarely** | **Occasionally** | **Most of the time** | **Always** |
| --- | --- | --- | --- | --- | --- |
| 1. Use consistent communication to discuss patient care. |  |  |  |  |  |
| 1. Coordinate health and social services (e.g., financial, occupational, housing) based upon patient care needs. |  |  |  |  |  |
| 1. Exercise shared decision-making capacity (e.g., responsibilities, decisions) with other health professionals. |  |  |  |  |  |
| 1. Respect and trust other health professionals. |  |  |  |  |  |
| 1. Am open and honest with other health professionals. |  |  |  |  |  |
| 1. Strive to achieve mutually satisfying resolution for differences of opinions. |  |  |  |  |  |
| 1. Understand the role boundaries of other health professionals. |  |  |  |  |  |
| 1. Understand that there are shared knowledge and skills between health professionals. |  |  |  |  |  |
| 1. Establish a sense of trust with other health professionals. |  |  |  |  |  |

**SECTION 8: COMMUNITY PARAMEDICINE PROGRAM CHARACTERISTICS**

*This section will ask questions about your community paramedicine program.*

***Instruction:*** *Please select the option that best corresponds with your program characteristics.*

1. **In what setting do you practice as a community paramedic?**

| Non-metropolitan | Small Rural (Less than 10,000 residents)   Large Rural (10,000 to 49,999 residents) |
| --- | --- |
| Metropolitan |  Small Metro (Less than 250,000 residents)   Medium Metro (250,000 to 999,999 residents)   Large Metro (1 million or more residents) |

1. **In what geographical region do you practice as a community paramedic?**

|  Northeast | Connecticut, Maine, Massachusetts, New Hampshire, Rhodes Island, Vermont, New Jersey, New York, Pennsylvania |
| --- | --- |
|  Midwest | Illinois, Indiana, Michigan, Ohio, Wisconsin, Iowa, Kansas, Minnesota, Missouri, Nebraska, North Dakota, South Dakota |
|  South | Delaware, Florida, Georgia, Maryland, North Carolina, South Carolina, Virginia, District of Columbia, West Virginia, District of Columbia, West Virginia, Alabama, Kentucky, Mississippi, Tennessee, Arkansas, Louisiana, Oklahoma, Texas |
|  West | Arizona, Colorado, Idaho, Montana, Nevada, New Mexico, Utah, Wyoming, Alaska, California, Hawaii, Oregon, Washington |

1. **How long has your community paramedic program been operational?**

 Less than 1 year

 1 to 2 years

 3 to 4 years

 5 years or more

1. **What type of delivery model is your community paramedicine program?**

**(Select all that apply)**

 Fire department

 Hospital-based

 Public - County

 Public - City

 Public - Regional

 Public Utility Model (government contract)

 Private (For-profit)

 Private (Nonprofit)

 Law enforcement

 Military

 Industrial

 Other (please specify): ______

1. **Which of the below best describes your patient population? (select all that apply)**

 Individuals with chronic conditions (e.g., hypertension, heart failure, diabetes, pneumonia)

 Individuals with disability

 Homeless individuals

 Individuals with mental health conditions

 Individuals with substance/alcohol abuse

 Uninsured individuals

 High EMS users

 High ED users

 Individuals in hospice care

 Older adults (≥ 65 years)

 Children

 Other (please specify) ______

1. **How is your program funded? (select all that apply)**

 Foundation/charitable grants

 Federal government

 State government

 Local government

 Insurance providers

 EMS departments

 Health care agencies (e.g., hospitals)

 Other (please specify) ______

 Don’t know

1. **Please indicate how you share data with other health professionals. (select all that apply)**

 Electronic health record systems (e.g., hospital or primary care provider systems)

 Health information exchange systems

 Encrypted email

 Faxing

 Telephone

 Manually (pen and paper)

 Other (please specify) _______

1. **Please indicate what outcomes are documented in your community paramedicine program (select all that apply).**

 Health services utilization

(e.g., hospital readmission/admissions, ED transport, ED visit, length of stays)

 Cost savings

 Patient clinical outcomes (e.g., blood pressure and blood glucose control)

 Patient-reported outcomes (e.g., patient satisfaction, health-related quality of life)

 Process measures (e.g., referrals, immunization)

 Other (please specify) ______

 Don’t know

1. **Do you practice as part of a mobile integrated health team?**

 Yes

 No

1. **Please indicate the type of mobile integrated health team (MIH) team that most closely represents your usual MIH operation.**

 Independent (I work by myself in collaboration with medical oversight)

 Pre-hospital (I work with another paramedic or EMT)

 Integrated (I work with another health care professional e.g., physician, nurse, social

worker)

 Other (please specify) ______

**SECTION 9: DEMOGRAPHIC & BACKGROUND CHARACTERISTICS**

*This section is comprised of questions about your background. Please fill in your response or select the option that best corresponds to your answer for each of the following questions.*

1. **What is your age?** ______ years
2. **With which gender identity do you most identify?**

 Male

 Female

 Non-binary

 Transgender Male

 Transgender Female

 Prefer Not to Answer

 Other (please specify) _______

1. **Which of the following best describes your race/ethnicity? (Select all that apply).**

 Non-Hispanic White

 Non-Hispanic Black

 Hispanic or Latinx

 American Indian or Alaska Native

 Native Hawaiian or Pacific Islander

 Asian

 Other (please specify) ______

1. **What is your highest educational level?**

 High school or GED

 Technical college certificate

 Associate degree

 Bachelor’s degree

 Master’s degree

 Other (please specify): __

1. **How many hours per week is allocated to your role as a community paramedic?**

______ hours per week

1. **How long have you worked as a community paramedic?**

 Less than 1 year

 1 year to 2 years

 3 years to 4 years

 Greater than 4 years

1. **Prior to your present role as a community paramedic, how long did you work as an EMT or paramedic in emergency care?**

______ years

**SECTION 10: FUTURE OF COMMUNITY PARAMEDICINE**

**Finally, we wish to obtain your viewpoint about the future of community paramedicine programs.**

1. **Where do you see your local community paramedicine program going in the next 3 – 5 years?**
2. **Where do you see the field of community paramedicine going in the next 10 – 20 years?**

*Your participation in this survey is appreciated.*

*If you would like to enter a drawing for the chance to win EMS medical gear, please enter your name and email address below.*

*Name:*

*Email:*

*If you do not wish to participate, simply click on the ‘Next’ option to end the survey.*

*This is the end of the survey. Thank you for participating.*
